# Biosignals Analysis for Kidney Function Effect Analysis of Fennel Aromatherapy

**DOI:** 10.1155/2015/267612

**Published:** 2015-04-21

**Authors:** Bong-Hyun Kim, Dong-Uk Cho, Ssang-Hee Seo

**Affiliations:** ^1^Department of Medical Electronics, Chungbuk Provincial University, 15 Daehak-ro, Okcheon-eup, Okcheon-gun, Chungcheongbuk-do 373-803, Republic of Korea; ^2^Department of Computer Engineering, Kyungnam University, 11 Woryeongbuk 16-gil, Masanhappo-gu, Changwon-si, Gyeongsangnam-do 631-701, Republic of Korea

## Abstract

Human effort in order to enjoy a healthy life is diverse. IT technology to these analyzes, the results of development efforts, it has been applied. Therefore, I use the care and maintenance diagnostic health management and prevention than treatment. In particular, the aromatherapy treatment easy to use without the side effects there is no irritation, are widely used in modern society. In this paper, we measured the aroma effect by applying a biosignal analysis techniques; an experiment was performed to analyze. In particular, we design methods and processes of research based on the theory aroma that affect renal function. Therefore, in this paper, measuring the biosignals and after fennel aromatherapy treatment prior to the enforcement of the mutual comparison, through the analysis, studies were carried out to analyze the effect of fennel aromatherapy therapy on kidney function.

## 1. Introduction

In modern society, efforts for health which has diversification number of methods have been used. Particularly, it tends to emphasize diet, health, and beauty and the quality of life among them, but they are used in a variety of ways, such as alternative medicine for health and diet. Alternative medicine is a medical application of the generic concept for the treatment of various ranges. It contains all preventive currently not recommended or not proven based on the evaluation of the clinician, diagnostic, and therapeutic methods. The alternative medicine is some diet, nutritional therapy, traditional therapy, private therapy, drug therapy, and herbal therapy. Aromatherapy therapy is a treatment that uses the scent by doing one of the many alternative medicines. In particular, scent therapy is called, as well as aromatherapy, aromatherapy therapy is a treatment in order to reduce stress and sedation of nerve, and smell the fragrance with the effect on the human body organs, acting on the brain.

Further, it is a therapeutic method in which the effectiveness in improving sleep using the herb is a plant which has been used since ancient times cosmetic and body various functions [1, 2]. Usually, it is widely used as a natural remedy for stress, but in the case of herbs and flowers in the direction of some there is also effectiveness in improving function of the human body organ which is known. The fennel herb has various effects; in this way there is a weight loss effect and activation of kidney function. When you drink in the car, improved digestive function shows the effect on menstrual irregularities and menstrual pain in women [2, 3].

Therefore, in this paper, in order to analyze the effects of herbs of fennel on the kidney function, an experiment was conducted by applying biosignal analysis techniques to measure the objective results for the treatment of fennel aromatherapy. Therefore, the herb of fennel is exposed by using the fragrance of fennel steam in confined spaces collecting the vital signs after fennel aromatherapy therapy before enforcement comparison of each other and was analyzed. Using the analytical parameter of image and voice signals that are related to kidney through the analysis of correlation between kidney function and fennel aromatherapy therapy biosignal analysis technique used in the study on the kidney herb fennel experimental results, I analyzed the impact.

## 2. Research Application Theory and Method

Theory of research that was applied in the present paper is the convergence theory of the biological signal analysis technology and diagnostic theory, aromatherapy therapy, and oriental medicine. Aromatherapy therapy refers to therapy fragrant aromatherapy in terms of combining the therapy and aroma. It is a natural therapy that uses pure essential oil 100% extracted from herbs plant fragrant purpose of improving health, disease prevention, beauty, and so forth. Aromatherapy has been passed down for thousands of years, is not described in the scientific methods of modern, and did not receive the interest, but its alternative medicine began the limelight from 20 to 30 years ago [4]. In particular, the fennel that has the effect on me to remove the toxin by too much drinking at the hub of aromatic herbs flower yellow letting you improve kidney function is known to fennel.

There are four diagnostic theories of Chinese medicine, in the paper, which were used in a composite manner auscultation theory to diagnose hearing the voice of the ocular inspection theory to be diagnosed by looking at the color and shape of the face. Ocular inspection theory was classified into five face area. It is a theory in which the liver is area of the left cheek, the heart is area of the forehead, the spleen is area of the face center, the lungs are area of the right cheek, and the kidney is area of the chin which is connected to the theory. That is in the face area a theory has been diagnosed color of the region turns there is a problem in the organs of the human body are connected. Therefore, in this paper, an experiment was conducted to analyze the correlation between kidney function and fennel through the change in color of the chin area [5–7].

Auscultation theory is a theory according to the voice and to determine the accuracy of pronunciation, diagnosing problems of body organs. In other words, liver is the sound of the back teeth, heart is the sound of the lingual, lungs are the sound of the teeth, and kidney is the sound of the lips that is connected to the theory (Table 1). Therefore, in this paper, by measuring the change in the sound of the lips, and analyzed, and the experiment the effect of fennel on kidney function [7, 8].

Finally, biomedical signal analysis technology is the application of the voice signal analysis technology and color analysis of the face area. Assay of the color of the face area was analyzed by changes in black through the lab color system by classifying after fennel aromatherapy before enforcement of the color chin region associated with the kidney [9]. Moreover, the experimental analysis of the speech signal compares the speech after fennel aromatherapy before enforcement based on the first formant frequency bandwidth associated with the sound of the lips and is analyzed.

## 3. Experimental and Result Analysis

In this paper, we conducted a study by applying the biosignal analysis techniques to analyze the effect of fennel aromatherapy on kidney function. Therefore, physiological signal collected each image and voice signals (Figure 2). Then, in the face area the image signal studied color analysis of the chin region associated with the kidney [10, 11]. Also, the voice signal experimented analysis of the first formant frequency bandwidth is associated with the kidney.

It collects face image signal and voice signal before and after the enforcement of fennel aromatherapy mutual comparison of the experimental method which was analyzed. In the course of the experiment, exposing to the scent of fennel in 100 minutes lab bench in a typical classroom compared to the image and voice signals before the experiment was analyzed.

### 3.1. Image Signal Analysis Experiment

**Figure 1:**
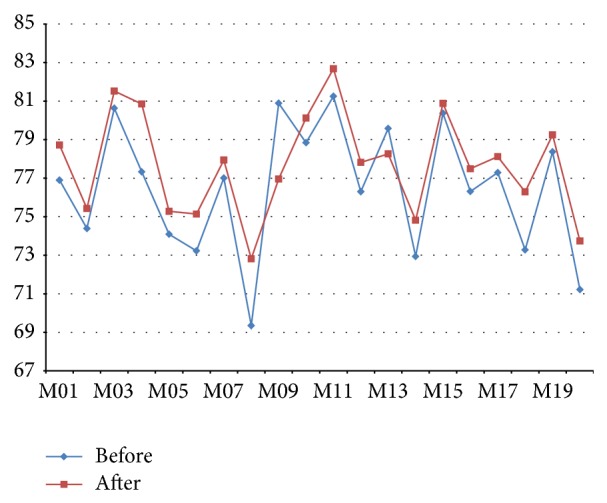
Image signals analysis result.

**Figure 2:**
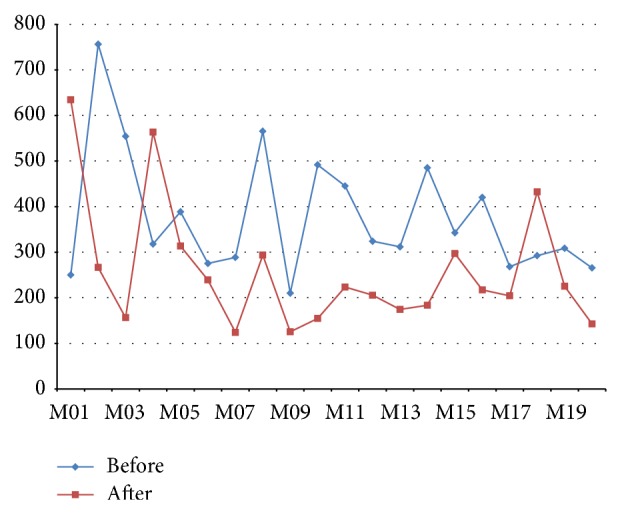
Voice signals analysis result.

Comparison of the color of the chin region using the lab color system analytical techniques image signals used in this paper is an analytical method. By using this, we analyzed the effect of fennel aromatherapy on kidney function (Figure 1).

In the experiment, collecting the face images before and after exposing to the fragrance of fennel targeting 20 men in their twenties was measured by the change in color of the chin region. For diagnostic theory of oriental medicine the color associated with kidney which is black was measured by *L* values of the chin region. The experimental environment shooting distance was set to 1.2 m using the lens of Tamron SP AF ASPHERICAL XR Di 28–75 mm and the camera body of Canon EOS-7D.Main text paragraph [11, 12].

Results of the experiment, after 90% of the subjects were exposed to the fragrance of fennel, *L* values of the chin area are increased. These results are analyzed; kidney function is improved temporarily by the smell of fennel; the value of *L* is increased. After all, it is analyzed that the aromatherapy of fennel can affect kidney function.

### 3.2. Voice Signal Analysis Experiment

The experimental analysis of the voice signal is based on oriental medicine auscultation theory, analyzing the sounds of the lips associated with kidney using the first formant frequency bandwidth. It means that the resonance of the latter formant is the formant frequency waveforms generated here. In addition, it appears that energy of a particular frequency band is aggregated and is also used to type the word sound board that is formants and this is the formant frequency bandwidth (Table 3). The experimental method is exposed to the fragrance of 100-minute fennel as in the image signal analysis experimental and compares it to collect the voice before and after exposure and is analyzed. Experiment environment uses the SONY ICD-SX67 high-quality recording mode STHQ method of stereo. At this time, fixed with 10 cm distance of subject and sound collection device you have to use the experimental sentence “Mapado Pyeongmin Pokpung Makmal” in the center is the sound of lips “ㅁ, ㅂ, and ㅍ” [13].

By the results of the analysis of the voice signal experiment, I found that, after 85% of all subjects underwent fennel aromatherapy, the first formant frequency bandwidth is reduced. Analyses of these results have affected temporarily improved kidney function by fennel aromatherapy (Table 2).

## 4. Conclusions

In this paper, aromatherapy of fennel studies was carried out to analyze the effects on kidney function based on the diagnostic theory of oriental medicine. Therefore, we performed analytical studies of the voice signal based on auscultation theoretical and experimental analysis of the face color of ocular inspection theoretical basis. In the experiment, the method was applied to collect each of the images and voice signals of the face of the post and fennel aromatherapy before enforcement comparison of each other and was analyzed. Regarding the experimental results and the analysis of the image signal, *L* values of the chin region of 90% of subjects associated with kidney increased. Also, by analysis of the voice signal, the first formant frequency bandwidth of 85% of subjects associated with kidney is reduced. It is determined that it may be possible to analyze a result, aromatherapy fennel to be effective in improving kidney function maintaining kidney function in aromatherapy fennel continuous and manage.

## Figures and Tables

**Table 1:** Five-parameter table.

Separation	Mok (wood)	Wha (fire)	To (soil)	Geum (iron)	Su (water)
Five viscera	Liver	Cardiac	Spleen	Lung	Kidney
Five colors	Blue	Red	Yellow	White	Black
Sounds	ㄱ, ㅋ	ㄴ, ㄷ, ㄹ, ㅌ	ㅇ, ㅎ	ㅅ, ㅈ, ㅊ	ㅁ, ㅂ, ㅍ
Pronunciation	Velar sound	Lingual sound	Guttural sound	Dental sound	Labial sound
Five sounds	Gak	Chi	Goong	Sang	Woo

**Table 2:** *L* value results of fennel aromatherapy.

	*L* value (lab digital color system)		
	Before	After	Deviation
M01	76.90	78.72	1.82
M02	74.39	75.43	1.04
M03	80.63	81.52	0.89
M04	77.33	80.85	3.52
M05	74.09	75.28	1.19
M06	73.23	75.14	1.91
M07	77.01	77.94	0.93
M08	69.35	72.82	3.47
M09	80.89	76.96	−3.93
M10	78.84	80.12	1.28
M11	81.25	82.68	1.43
M12	76.30	77.82	1.25
M13	79.58	78.26	−1.32
M14	72.94	74.82	1.88
M15	80.37	80.88	0.51
M16	76.31	77.49	1.18
M17	77.29	78.12	0.83
M18	73.28	76.29	3.01
M19	78.37	79.25	0.88
M20	71.22	73.74	2.52

**Table 3:** The first formant frequency bandwidth results of fennel aromatherapy.

	The first formant frequency bandwidth		
	Before	After	Deviation
M01	250.248	634.285	384.037
M02	756.384	266.541	−489.843
M03	554.295	156.394	−397.901
M04	317.920	563.482	245.562
M05	388.681	313.520	−75.161
M06	275.394	239.054	−36.340
M07	288.658	124.259	−164.399
M08	565.337	293.560	−271.777
M09	210.118	125.406	−84.712
M10	491.624	154.672	−336.952
M11	445.284	223.541	−221.743
M12	324.105	205.842	−118.263
M13	311.826	174.527	−137.299
M14	485.267	183.605	−301.662
M15	342.691	297.058	−45.633
M16	420.183	217.259	−202.924
M17	268.254	204.253	−64.001
M18	292.406	432.524	140.118
M19	308.652	225.367	−83.285
M20	265.485	142.630	−122.855
